# Deep brain stimulation of the nucleus accumbens in treatment-resistant alcohol use disorder: a double-blind randomized controlled multi-center trial

**DOI:** 10.1038/s41398-023-02337-1

**Published:** 2023-02-08

**Authors:** Patrick Bach, Mathias Luderer, Ulf Joachim Müller, Martin Jakobs, Juan Carlos Baldermann, Jürgen Voges, Karl Kiening, Anke Lux, Veerle Visser-Vandewalle, the DeBraSTRA study group, Joachim Klosterkötter, Daniel Huys, Wolfgang Sommer, Tillmann Weber, Bernhard Bogerts, Jens Kuhn, Karl Mann

**Affiliations:** 1 Department of Addictive Behavior and Addiction Medicine, Central Institute of Mental Health, Medical Faculty Mannheim, Heidelberg University, Mannheim, Germany; 2 Feuerlein Center on Translational Addiction Medicine (FCTS), University of Heidelberg, Heidelberg, Germany; 3 Department of Psychiatry, Psychosomatic Medicine and Psychotherapy, University Hospital, Goethe University, Frankfurt, Germany; 4 Department of Psychiatry and Psychotherapy, University Hospital, Otto-v.-Guericke University, Magdeburg, Germany; 5 Division for Stereotactic Neurosurgery, Department of Neurosurgery, University Hospital Heidelberg, Heidelberg, Germany; 6 Department of Psychiatry and Psychotherapy, Faculty of Medicine and University Hospital Cologne, University of Cologne, Cologne, Germany; 7 Department of Neurology, Faculty of Medicine and University Hospital Cologne, University of Cologne, Cologne, Germany; 8 Department of Stereotactic Neurosurgery, University Hospital, Otto-v.-Guericke University, Magdeburg, Germany; 9 Institute of Biometry and Medical Informatics of the University Hospital Magdeburg, Magdeburg University, Magdeburg, Germany; 10 Department of Stereotactic Neurosurgery, University Hospital Cologne, Cologne, Germany; 11 Department of Psychiatry, Psychotherapy and Psychosomatics, Johanniter Hospital, Oberhausen, Germany; 12 Institute of Psychopharmacology, Central Institute of Mental Health, Medical Faculty Mannheim, Heidelberg University, Heidelberg, Germany; 13 AHG hospital Wilhelmsheim, Wilhelmsheim, Germany

**Keywords:** Addiction, Neuroscience

## Abstract

Treatment resistance in alcohol use disorders (AUD) is a major problem for affected individuals and for society. In the search of new treatment options, few case studies using deep brain stimulation (DBS) of the nucleus accumbens have indicated positive effects in AUD. Here we report a double-blind randomized controlled trial comparing active DBS (“DBS-EARLY ON”) against sham stimulation (“DBS-LATE ON”) over 6 months in *n* = 12 AUD inpatients. This 6-month blind phase was followed by a 12-month unblinded period in which all patients received active DBS. Continuous abstinence (primary outcome), alcohol use, alcohol craving, depressiveness, anxiety, anhedonia and quality of life served as outcome parameters. The primary intention-to-treat analysis, comparing continuous abstinence between treatment groups, did not yield statistically significant results, most likely due to the restricted number of participants. In light of the resulting limited statistical power, there is the question of whether DBS effects on secondary outcomes can nonetheless be interpreted as indicative of an therapeutic effect. Analyses of secondary outcomes provide evidence for this, demonstrating a significantly higher proportion of abstinent days, lower alcohol craving and anhedonia in the DBS-EARLY ON group 6 months after randomization. Exploratory responder analyses indicated that patients with high baseline alcohol craving, depressiveness and anhedonia responded to DBS. The results of this first randomized controlled trial are suggestive of beneficial effects of DBS in treatment-resistant AUD and encourage a replication in larger samples.

## Introduction

Alcohol use disorder (AUD) is among the most frequent and devastating diseases globally [1]. Currently approved pharmacological and non-pharmacological treatments leave much room for improvement. Even when treated according to current guidelines, the majority of AUD patients relapse within a short period [2]. New treatments are needed, especially for the group of treatment-resistant AUD patients who respond poorly to currently available treatment options [3]. Treatment-resistant AUD has been associated with increased incentive sensitization to alcohol-associated cues, accompanied by attentional bias towards such cues, as well as increased craving [4]. Regarding the neurobiological basis of these processes, animal models demonstrated that ethanol intake and the anticipation of ethanol both trigger a phasic dopamine release in the Nucleus accumbens (NAc). This dopamine release, in turn, modulates activation in the mesocorticolimbic system implicated in craving, reward and behavioral control [5–8]. Although a wide range of brain areas have been implicated in the development and maintenance of addictive behaviors, the NAc has been identified as a key region in animal and human models of AUD [9, 10]. Past neuroimaging studies have uncovered evidence of alcohol-cue exposure increasing brain activation in the NAc and striatum [11–14]. Brain activation in these areas has been repeatedly demonstrated to be correlated to subjective alcohol craving [11, 15], to predict relapse in AUD patients after withdrawal treatment [16, 17] and to be associated with a better treatment response to naltrexone, an opioid antagonist [18–20].

In recent years efforts have been undertaken to develop innovative interventions to modulate NAc activation and assess the clinical effects of such interventions. One such intervention is Deep Brain Stimulation (DBS). DBS is an approved treatment for several movement disorders (e.g. Parkinson’s disease, tremor, and dystonia) and epilepsy in various brain targets and has been used in clinical practice for more than 25 years [21]. Under its Human Device Exemption, the U.S. Food and Drug Administration has approved of DBS targeting the anterior limb of the internal capsule (ALIC) neighboring the NAc as an option for treating patients with refractory obsessive-compulsive disorder (OCD) [22]. Initial translational studies on DBS and optogenetic stimulation of the NAc have yielded promising results in animal models of addiction [23–26].

Using DBS to treat addiction in humans has been documented in the literature for more than 15 years. It can be traced back to a first incidental finding of a patient with severe treatment-resistant anxiety disorder and comorbid AUD consuming much less alcohol while being treated with NAc stimulation [27]. In the following years, several human studies and case reports have investigated the effects of DBS in various substance-use disorders. In AUD results from nine patients altogether have been published to date [27–31]. A recent open-label study in six AUD patients provided further support for the beneficial effects of DBS in AUD [32]. Patients reported a reduction in alcohol consumption, alcohol craving, and anxiety after 12 months of stimulation. In addition, reduced NAc metabolism during positron emission tomography (PET) studies and a reduced NAc activation and connectivity during functional magnetic resonance imaging (fMRI) were observed. Even though these studies reported positive effects, open-label, non-randomized study designs leave open the question of whether or not the observed effects are attributable to DBS. In the intention to close this gap, we conducted a randomized controlled double-blind multi-center trial (“Deep Brain Stimulation in Treatment-Resistant Alcoholism” - DeBraSTRA) investigating the effects of NAc DBS in a sample of heavily dependent AUD patients over a period of 18 months.

## Methods and materials

### Study design and procedures

**Fig. 1:**
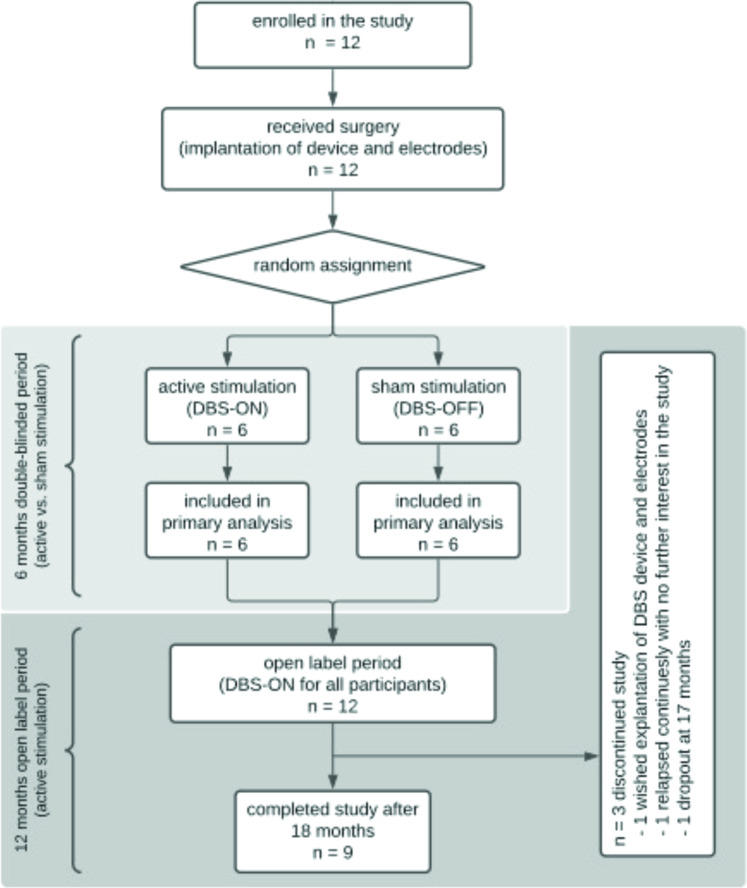
Study Flow-chart. Of the 30 planned patients, only 12 patients were ultimately enrolled (*n* = 4 per center) (2013 – 2016). One reason for the lower-than-expected inclusion rate was the strict inclusion and exclusion criteria, which followed those used in previous studies and were approved by the applicable ethics committees. For the primary outcome “time to first alcohol use”, data was available for all *n* = 12 participants from baseline until the end of the blinded study period at month 6 (100%). For the secondary outcomes, data was available at baseline for all participants and for 9 participants at months 6 and 18. After 18 months, 75% of included participants (*n* = 9) had completed the study.

The study was preregistered (German clinical trials database: DRKS00003206, EUDAMED ID: CIV-11-05-000663) and conducted in three German centers (Cologne, Magdeburg, and Mannheim/Heidelberg) as a double-blind randomized controlled trial (RCT). After baseline assessment and randomization all participants received bilateral stereotactic implantation of DBS electrodes in the NAc with sub-clavicular implantation of the pulse generator (see Supplements for details on the surgical procedure and Supplementary Fig. 1). After surgery, patients were randomized to either receive sham (DBS-LATE ON) or active stimulation (DBS-EARLY ON) during the first 6 months of the study. The first 6 months of active or sham stimulation were followed by a 12-month open-label period of active stimulation in all patients (see Fig. 1). Safety and outcome measures were assessed for a total of 18 months (see Fig. 1). A block-wise randomization was conducted within the study centers with a randomly varied block size between 2 and 4. Both, clinical investigators and patients were blinded to whether patients received active treatment or not.

The trial was conducted in accordance to Good Clinical Practice guidelines (ICH-GCP) and the Declaration of Helsinki. All study procedures were approved by the German Federal Institute for Drug Safety and the German Federal Office for Radiation Protection as well as the local ethics committees of all study centers. The Institute of Biometry and Medical Informatics of the University Hospital Magdeburg provided biometrical support for the RCT. Independent and steady monitoring was provided by the coordination center for clinical studies of the University of Magdeburg and by an external data safety and monitoring board.

### Surgical procedure and stimulation parameters

The surgical procedure followed a predefined protocol in all study centers. Treatment planning for stereotactic bilateral implantation of quadripolar DBS-brain electrodes (3387, Medtronic, Minneapolis, MN, USA) was based on high-resolution pre- or intraoperative MRI images (1.5T or 3.0T MRI-scanner). The target for electrode implantation (translating to the inferior border of the most distal electrode contact) was defined by a combination of indirect coordinate-based and direct anatomical targeting. Standard coordinates in relation to the midsagittal plane were: *X* = 6–8 mm lateral to the midline, *Y* = 2 mm rostral to the anterior border of the anterior commissure, and *Z* = 3–4 mm ventral [33, 34]. From here targeting was refined by anatomical means. In coronal MRI images (T1 or proton density weighted T2 images) the slice that most clearly outlined Broca’s diagonal band (a gray matter tract encapsulating the NAc medially and inferiorly) was chosen. Herein the target was defined to be 2.0–2.5 mm lateral to the vertical limb of Broca’s diagonal band and dorsal to the Tuber olfactorius. A deep frontolateral position for the entry point in relation to the coronal suture was used to enable a trajectory running parallel to and inside the ALIC. Targeting this way aimed at placing the two distal contacts of the DBS-electrode into the caudo-medial NAc, the third contact at the transitional border between the NAc and the ALIC and the fourth contact into the most ventral part of the ALIC or in transition to the Caudate nucleus.

**Fig. 2:**
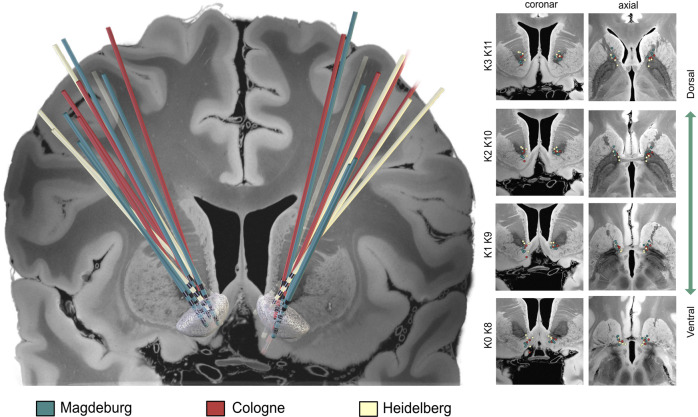
Overview of electrode localization in standard space. All electrodes, colored by center, were reconstructed from preoperative magnetic resonance imaging and postoperative computer tomography, following the default pipeline of the LEAD-DBS software [75]. In the left image, the target area, i.e. the nucleus accumbens, is depicted as white mesh. The right image displays the projection on 2D slices for each contact level. All images are superimposed on slices of a 7 Tesla brain scan in MNI space [76].

Both, frame-based stereotactic implantation of the brain electrodes and implantation of the non-rechargeable pulse generator (ACTIVA®PC, Medtronic; Minneapolis, MN, USA) in the subclavicular area were performed as a single surgery under general anesthesia. All patients underwent post- or intraoperative CT- or MRI scans for verification and documentation of final electrode placement (see Fig. 2 for depiction of electrode localizations) and to exclude intracerebral hemorrhage. All study participants were treated as inpatients for at least one week after surgery.

Initial stimulation parameters were empirically chosen and carried over from the previous case studies and series of NAc DBS for psychiatric disorders under which patients experienced significant improvement of symptoms without encountering any stimulation-induced side effects [35, 36]. Stimulation was started at 130 Hz, 90 µs, and 3.5 V with the two most distal contacts being activated for cathodic monopolar stimulation aiming to cover a large portion of the NAc volume. Adjustment of the stimulation amplitude by 0.5 V each (culminating in a maximum amplitude of 4.5 V) could be performed at 2 and 4 weeks after initiating stimulation (both after baseline in the DBS-EARLY ON group and after 6 months in the DBS-LATE ON group) if no stimulation-induced side effects were observed or suboptimal treatment response was seen during a standardized interview. Patients were evaluated for changes in mood, calmness, anxiety, and craving. Other changes in stimulation parameters were not permitted.

### Study visits

Study visits were conducted at baseline, in weeks 2, 4, 6, 8 and then monthly. Visits included assessment of adverse events and primary and secondary outcome measures. Alcohol use and abstinence were assessed through patient reports using the validated Form-90 interview [37]. Alcohol craving was measured using the Obsessive-Compulsive-Drinking-Scale (OCDS), which assesses alcohol craving over the last seven days [38, 39] and the Alcohol Urge Questionnaire (AUQ) [40], which assesses momentary craving. Depressive symptoms were investigated using the Beck Depression Inventory (BDI-II) [41] and the Hamilton Depression Scale (HAMD) [42]. In addition, anhedonia was assessed using the Snaith-Hamilton-Pleasure-Scale (SHAPS) [43] and the Chapman Anhedonia Scale [44]. The Young Mania Rating scale was used to assess euphoria and symptoms of (hypo-)mania throughout the trial [45]. For Quality of Life we used the German version of the World Health Organization’s quality of life questionnaire (WHOQOL-BREF) [46]. Psychosocial functioning was assessed using the Global Assessment of Functioning scale (GAF) [47]. Adverse events were recorded at every study visit and between study visits, if reported by the participants. Before study inclusion, after electrode implantation and at 6 months follow-up (end of sham-stimulation) a full neurological and psychiatric examination was conducted and blood samples were drawn to determine indirect measures of alcohol use (e.g., Carbohydrate Deficient Transferrin). Control of the stimulation parameters was done by an unblinded researcher at each site who was independent and not involved in any other assessment.

### Participants

The local ethics committees requested only male AUD patients to be included into the trial. Patients were enrolled if they met all following inclusion criteria: (i) age between 25 and 60 years, (ii) diagnosis of alcohol dependence for at least 10 years according to the Diagnostic and Statistical Manual of Mental Disorders 4th revision (DSM-IV-TR) and the International Classification of Disease 10th revision (ICD-10), (iii) completion of at least 9 years of school education, (iv) capacity to understand study procedures and provide informed written consent, (v) meet criteria of “treatment resistance”. This was defined as alcohol dependence for at least 10 years, at least 2 inpatient or day-care rehabilitation treatments of a total duration of at least 6 months and unsuccessful treatment with acamprosate or naltrexone, and (vi) consumption of at least 30 standard drinks (14 grams pure alcohol) per week over a 30-day period within the last 3 months.

Exclusion criteria were: (i) abuse of or addiction to other substances (other than nicotine and alcohol) with a positive urine screening, (ii) other DSM-IV-TR axis-I disorders (excluded via a full SCID-I interview [48]), (iii) antisocial personality disorder (i.e. score >20 on the psychopathy check list [49]), (iv) brain damage, diagnosed on MRI-scan by a neuroradiologist, v) severe neurological or medical conditions, as well as vi) any general contraindication for surgery and/or anesthesia.

The inclusion and exclusion criteria matched those used during previous trials [29]. During the three years of recruitment (2013-2015), a total of about 750 patients were treated at the three study sites with a diagnosis of an alcohol addiction or alcohol-related disease (ICD-10: F10.2, F10.3 and F10.4) and a duration of inpatient treatment at least one week. Most of these patients however did not meet the strict inclusion and exclusion criteria. Specifically, the majority of these patients did either not meet the inclusion criterion of treatment resistance (see above) or presented with a psychiatric (e.g., other addiction diagnoses) or somatic comorbidity. In addition, a relevant proportion of patients declined to participate in the study because of the surgical procedure involved.

Because of medical detoxification as inpatients, all participants were abstinent at the time of surgery.

### Primary outcome

Continuous abstinence from alcohol, i.e. the time to first alcohol use within the first 6 months after randomization served as the primary outcome and was assessed using the validated Form-90 interview [37]. The choice of the primary outcome followed German treatment guidelines and recommendations by the European Medicines Agency (EMA/CHMP/EWP/20097/2008).

### Secondary Outcomes

1. Alcohol consumption (i.e. proportion of abstinent days, proportion of heavy drinking days, mean daily alcohol use) during the 6 months after randomization (blinded phase) and the following 12 months (unblinded ON phase).
2. Alcohol craving, depressive symptoms, anhedonia, anxiety, quality of life, and global functioning at months 6 and 18 after randomization.
3. Safety was assessed by means of occurrence of any adverse or serios adverse events throughout the 18-month study period.

### Hypotheses

We hypothesized that DBS-EARLY ON versus DBS-LATE ON results in a longer abstinence from alcohol during the first 6 months after randomization (primary outcome). Secondary hypotheses: DBS-EARLY ON versus DBS-LATE ON results in lower relative proportions of drinking days and heavy drinking days, as well as mean alcohol use at month 6 after randomization.

### Statistical analyses and sample size estimation

The primary analysis included all randomized patients, following an intention-to-treat principle. The primary endpoint was compared between both treatment arms using a log-rank test. For the power analyses we assumed a relapse rate (i.e. any alcohol use) of 90% in the DBS-LATE ON group vs. a relapse rate of 67% in the DBS-EARLY ON group, according to previous studies [27–30]. Sample sizes estimation indicated that *n* = 13 patients per group yield a power of 80% for a log-rank-test (*α* = 0.05, two-sided) comparing time to first alcohol use between both arms. To account for drop-outs, we aimed to recruit *n* = 15 patients per group, in order to yield a sample size of *n* = 30. The effect of DBS-EARLY ON was further quantified as hazard ratio (HR) using a proportional hazards Cox-regression model with 95% confidence interval. Secondary endpoints were compared between DBS-EARLY ON and DBS-LATE ON at month 6 after study randomization using Mann-Whitney U-tests. Within-group differences between baseline and after 6 and 18 months were compared using Wilcoxon-matched-pairs signed-rank tests. Furthermore, we conducted exploratory responder analyses to identify patient characteristics linked to DBS efficacy. Since a reduction of alcohol use by at least two WHO risk-drinking levels has been identified as an important outcome in clinical AUD trials by the European Medicines Agency (EMA) and has been substantiated by multiple clinical studies [50–53], we classified patients as responders (i.e. reduction at least two WHO risk drinking levels) and non-responders (i.e. reduction of less than two WHO risk-drinking levels) accordingly, based on the trajectories of their alcohol use from baseline to the last study visit (month 18).

## Results

A total of 12 patients were enrolled in the trial (*n* = 4 per center). For the primary outcome, data was available for all participants. For the secondary outcomes, data was available at baseline for all subjects and at months 6 and 18 for 9 participants (see Fig. 1). At baseline, there were no significant differences between the DBS-EARLY ON and DBS-LATE ON groups with the exception of a higher physical anhedonia score in the DBS-LATE ON group (see Table 1).

**Table 1:** Baseline data on demographic characteristics, alcohol use and severity measures for patients randomized to the active stimulation (DBS-EARLY ON) and sham stimulation (DBS-LATE ON) groups.

	DBS-EARLY ON	DBS-LATE ON	Statistics	Significance
	(*n* = 6)	(*n* = 6)		
Demographics	Mean (SD)	Mean (SD)		
Age (years)	44.2 (9.9)	47.7 (5.8)	*t*_(10)_ = 0.747	*p* = 0.472
Sex (%males)	100	100	–	–
Employed (yes/no)	1/5	2/4	–	–
Education (up to 9 years schooling/9–12 years schooling/12 or more years of schooling)	1/5/0	2/3/1	–	–
Marital status (married or partnered/divorced/single)	3/2/1	4/0/1^a^	–	–
Substance use patterns				
Ethanol (g/day, mean of last 90 days)	187.8 (144.9)	227.0 (189.6)	*U* = 13	*p* = 0.485
Drinks per day (à 12 g, mean of last 90 days)	15.6 (12.1)	18.9 (15.8)	*U* = 13	*p* = 0.485
Abstinent days (% of last 90 days)	33.0 (9.4)	22.3 (11.7)	*U* = 8	*p* = 0.132
Abstinence before randomization (days)	20.5 (8.2)	16.3 (8.1)	*U* = 16	*p* = 0.784
Smokers (*n*)	5 (100%^a^)	5 (83%)	-	-
Clinical scales				
AUDIT (sumscore)	32.5 (3.6)	32.7 (4.4)	*U* = 18	*p* = 1.000
ADS (sumscore)	26.3 (11.2)	20.7 (5.8)	*U* = 14	*p* = 0.563
OCDS (sumscore)	19.3 (5.1)	22.6 (8.7)	*U* = 15	*p* = 0.699
AUQ (sumscore)	16.2 (12.9)	25.2 (14.3)	*U* = 10	*p* = 0.210
FTND (sumscore)^a^	6.8 (1.3)	5.8 (3.7)	*U* = 14.5	*p* = 0.955
HAMD (sumscore)	3.7 (4.1)	0.5 (0.8)	*U* = 12	*p* = 0.318
BDI-II (sumscore)	10.0 (4.3)	12.5 (9.0)	*U* = 15	*p* = 0.738
SHAPS (sumscore)	3.8 (3.2)	3.0 (2.5)	*U* = 14.5	*p* = 0.602
Physical Anhedonia (sumscore)	4.5 (3.0)	7.5 (3.3)	*U* = 5.5	*p* = 0.039*
Social Anhedonia (sumscore)	8.5 (5.8)	11.0 (4.3)	*U* = 10	*p* = 0.234
HAM-A (sumscore)	2.0 (1.9)	2.5 (3.8)	*U* = 16.5	*p* = 0.840
WHOQOL-BREF (global domain)	46.0 (28.9)	39.6 (10.0)	*U* = 15.5	*p* = 0.736
GAF (sumscore)	54.2 (14.1)	55.5 (11.1)	*U* = 17	*p* = 0.907

*ADS* Alcohol Dependence Scale, *AUQ* Alcohol Urge Questionnaire; *AUDIT* Alcohol Use Disorders Identification Test, *BDI-II* Beck Depression Inventory, *FTND* Fagerstroem Test for Nicotine Dependence, *GAF* Global Assessment of Functioning Scale, *HAMD* Hamilton Depression Scale, *HAM-A* Hamilton Anxiety Rating Scale, *OCDS* Obsessive-Compulsive Drinking Scale, *SHAPS* Snaith-Hamilton Pleasure Scale, *STAI* State-Trait-Anxiety Inventory, *SD* standard deviation, *WHOQOL-BREF* World Health Organization Quality of Life Questionnaire, *U* Mann-Whitney *U* test.

*Significant differences between groups with *p* < 0.05.

^a^*n* = 1 patient chose to make no specification.

### Primary outcome parameter – time to first alcohol use

**Fig. 3:**
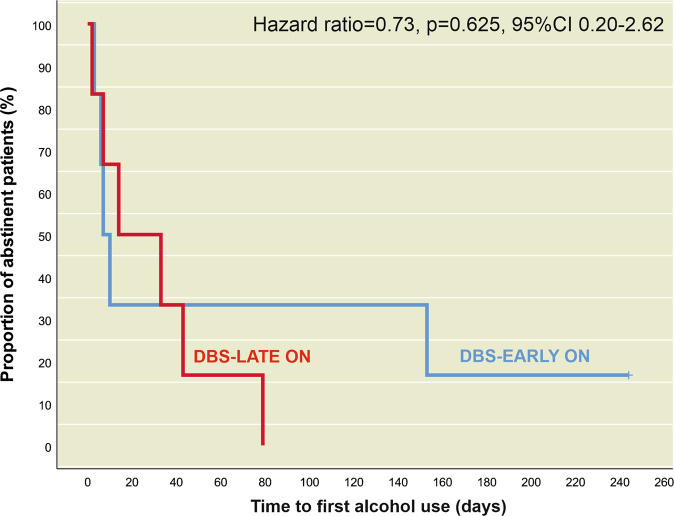
Effect of NAc DBS on time to first alcohol use. Kaplan–Meier curves illustrating the time until first alcohol use after randomization (primary outcome) in the active stimulation group (DBS EARLY-ON) and sham stimulation group (DBS LATE-ON), which did not differ significantly between both groups (95%CI = 95% Confidence Interval).

Overall, 11 out of 12 patients used alcohol during the first 6 months after randomization. In the DBS-EARLY ON group, one patient remained abstinent throughout the study and one patient remained abstinent for 5 months, while all patients in the DBS-LATE ON group had used alcohol by the end of month 6. The mean time to first alcohol use was 70.5 days (DBS-EARLY ON) and 29.7 days (DBS-LATE ON). There was no significant difference in terms of time to first alcohol use between the DBS-LATE ON and DBS-EARLY ON groups after the first 6 months of treatment (two-sided Log-Rank-Test, *p* = 0.619, see Fig. 3). Cox regression revealed a non-significant reduction in alcohol use risk for the DBS-EARLY ON group (HR = 0.73; 95%CI 0.20–2.62; *p* = 0.625). This HR would translate to a number needed to treat (NNT) of 9.6 [95%CI 1.9–16.4] [54]. The results remained unchanged when dependence severity and recency of alcohol use were included as covariates in the Cox regression model.

### Secondary outcomes

#### Alcohol use

**Fig. 4:**
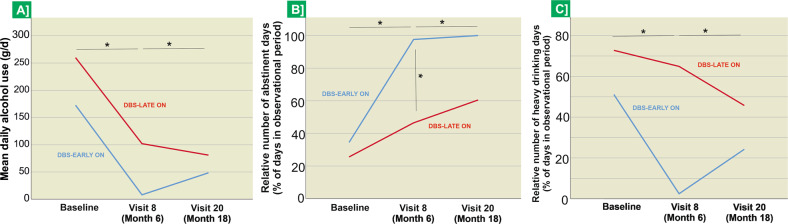
Effect of NAc DBS on alcohol use, abstinent days and heavy drinking days. Depiction of median values and of significant longitudinal changes and group differences in **A** mean alcohol use (mean over last 30 days), **B** proportion of abstinent days, and **C** the proportion of heavy drinking days, at baseline, at the 6-month visit (i.e., end of blinded phase), and at 18 months (after all patients had been actively stimulated for at least 12 months). *Significant differences between time points or study groups, as indicated, at *p* < 0.05, determined using Wilcoxon tests and Mann–Whitney *U* tests.

In accordance with the study protocol, the comparison of both study groups at month 6 after randomization revealed a significantly higher proportion of abstinent days in the DBS-EARLY ON group (*p* = 0.048, see Table 2A). Analyses of the means of the secondary outcome variables, calculated across the eight study visits that took place over the 6 months following randomization, corroborated this finding. Here, the DBS-EARLY ON group showed a significantly higher mean proportion of abstinent days (*p* = 0.032, see Table 2B and Fig. 4B) and fewer heavy drinking days (*p* = 0.041, see Table 2B and Fig. 4C). Comparison of other alcohol-use indices showed a lower mean alcohol consumption per day in the DBS-EARLY ON group (see Fig. 4A), although this comparison was not statistically significant. Comparing both groups at 18 months after randomization, i.e. after all patients had been stimulated for 12 months following the end of the double-blind study phase, revealed no significant differences between both study groups (all *p* values ≥ 0.05; see Table 2C). This descriptive observation was mirrored by longitudinal analyses in the whole study group and in both groups separately, comparing baseline against study visits 8 after 6 months (6 M) and 20 after 18 months (18 M). DBS treatment resulted in a significantly higher proportion of abstinent days at the end of the study (*n* = 9, baseline: 27.6% ± 11.6, 18 M: 74.2% ±31.3, *Z* = −2.547, *p* = 0.004) and lower proportion of heavy drinking days (*n* = 6, baseline: 69.6% ± 12.6, 18 M: 35.5% ± 29.9, *Z* = −1.992, *p* = 0.031), as well as a lower mean daily alcohol use (*n* = 9, baseline: 207.4 g/d ± 162.2, 18 M: 62.7 g/d ± 72.5, *Z* = −1.836, *p* = 0.037, see Fig. 4). A significant increase in the proportion of abstinent days was also observed when comparing baseline against the 6-month visit across both study groups (*n* = 12, baseline: 27.6% ± 11.6, 6 M: 56.0% ±31.1, *Z* = −2.191, *p* = 0.014), as well as a significant decrease in the proportion of heavy drinking days (*n* = 9, baseline: 69.6% ± 12.6, 6 M: 47.4% ± 27.6, *Z* = −2.073, *p* = 0.020) and mean daily alcohol use (*n* = 9, baseline: 207.4 g/d ± 162.2, 6 M: 85.3 g/d ± 69.7, *Z* = −2.192, *p* = 0.014, see Fig. 4).

**Table 2:** Results of the comparison between the DBS-EARLY ON and DBS-LATE ON groups on alcohol use variables, psychometric measures of depression, anxiety, anhedonia and quality of life (A) at 6 months after randomization (i.e. study visit 8), as per the original study specification, (B) over all *n* = 8 study visits during the first 6 months after randomization (cumulated mean values), and (C) at 18 months after randomization (i.e. study visit 20), as per the original study specification.

	DBS-EARLY ON	DBS-LATE ON	Statistics	Significance
	(*n* = 6)	(*n* = 6)		
(A) Study visit 8 (6 months after randomization)	Mean (SD)	Mean (SD)		
Substance use patterns				
Ethanol (g/day; mean of last 30 days)	72.9 (89.1)	102.6 (44.3)	*U* = 8	*p* = 0.365
Drinks per day (à 12 g, mean of last 30 days)	6.1 (7.4)	8.5 (3.7)	*U* = 8	*p* = 0.365
Abstinent days (% in last 30 days)	71.0 (32.9)	40.8 (22.7)	*U* = 4	*p* = 0.048*
Heavy Drinking days (>60 g/d, % in last 30 days)	35.1 (33.6)	57.2 (20.2)	*U* = 5	*p* = 0.143
Clinical scales				
OCDS (sumscore)	13.0 (11.0)	22.4 (7.8)	*U* = 15	*p* = 0.699
AUQ (sumscore)	11.2 (4.1)	23.4 (10.0)	*U* = 2.5	*p* = 0.020*
FTND (sumscore)^a^	6.8 (2.1)	6.5 (2.4)	*U* = 7	*p* = 0.500
HAMD (sumscore)	4.0 (5.0)	8.0 (7.4)	*U* = 8	*p* = 0.206
BDI-II (sumscore)	8.6 (7.1)	14.5 (11.1)	*U* = 6.5	*p* = 0.238
SHAPS (sumscore)	0.8 (1.1)	6.2 (5.4)	*U* = 3	*p* = 0.028*
Physical Anhedonia (sumscore)	5.8 (3.6)	8.2 (2.1)	*U* = 6	*p* = 0.198
Social Anhedonia (sumscore)	7.0 (3.2)	15.8 (3.4)	*U* = 0	*p* = 0.008*
HAM-A (sumscore)	5.2 (7.9)	5.2 (4.3)	*U* = 9.5	*p* = 0.294
WHOQOL-BREF (global domain)	55.0 (20.9)	48.9 (29.1)	*U* = 9	*p* = 0.452
GAF (sumscore)	63.3 (20.7)	52.8 (6.3)	*U* = 5.5	*p* = 0.314
(B) Study visits 1 to 8 (cumulated mean values across all study visits through month 6)				
Substance use patterns				
Ethanol (g/day; mean of last 30 days)	43.6 (56.2)	67.4 (46.8)	*U* = 12.5	*p* = 0.209
Drinks per day (à 12 g, mean of last 30 days)	3.6 (2.7)	5.6 (7.1)	*U* = 12.5	*p* = 0.209
Abstinent days (% in last 30 days)	80.4 (25.8)	50.0 (20.0)	*U* = 6	*p* = 0.032*
Heavy Drinking days (>60 g/d, % in last 30 days)	22.7 (26.7)	46.8 (22.5)	*U* = 5	*p* = 0.041*
(C) Study visit 20 (18 months after randomization)				
Substance use patterns				
Ethanol (g/day; mean of last 30 days)	72.9 (89.1)	102.6 (44.3)	*U* = 8	*p* = 0.357
Drinks per day (à 12 g, mean of last 30 days)	6.1 (7.4)	8.5 (3.7)	*U* = 8	*p* = 0. 357
Abstinent days (% in last 30 days)	71.0 (32.9)	40.8 (22.7)	*U* = 5	*p* = 0.143
Heavy Drinking days (>60 g/d, % in last 30 days)	35.1 (33.6)	57.2 (20.2)	*U* = 2	*p* = 0.267
Clinical scales				
OCDS (sumscore)	9.75 (10.3)	22.4 (7.8)	*U* = 15	*p* = 0.699
AUQ (sumscore)	8.3 (0.6)	27.8 (23.1)	*U* = 4	*p* = 0.286
FTND (sumscore)^a^	5.0 (1.0)	6.0 (2.6)	*U* = 4	*p* = 0.500
HAMD (sumscore)	1.0 (1.0)	8.3 (13.6)	*U* = 4	*p* = 0.500
BDI-II (sumscore)	3.8 (6.9)	9.7 (10.0)	*U* = 4	*p* = 0.286
SHAPS (sumscore)	0.8 (1.5)	3.7 (4.7)	*U* = 3.5	*p* = 0.257
Physical Anhedonia (sumscore)	5.8 (2.8)	7.7 (0.6)	*U* = 3.5	*p* = 0.257
Social Anhedonia (sumscore)	9.3 (3.7)	10.7 (5.1)	*U* = 4.5	*p* = 0.343
HAM-A (sumscore)	4.0 (4.0)	4.7 (5.0)	*U* = 4	*p* = 0.500
WHOQOL-BREF (global domain)	54.2 (36.1)	62.5 (21.7)	*U* = 4	*p* = 0.500
GAF (sumscore)	71.7 (26.6)	54.5 (22.8)	*U* = 2	*p* = 0.144

*ADS* Alcohol Dependence Scale, *AUQ* Alcohol Urge Questionnaire, *AUDIT* Alcohol Use Disorders Identification Test, *BDI-II* Beck Depression Inventory, *FTND* Fagerstroem Test for Nicotine Dependence, *GAF* Global Assessment of Functioning Scale; *HAMD* Hamilton Depression Scale, *HAM-A* Hamilton Anxiety Rating Scale, *OCDS* Obsessive-Compulsive Drinking Scale, *SHAPS* Snaith-Hamilton Pleasure Scale, *STAI* State-Trait-Anxiety Inventory, *SD* standard deviation; *WHOQOL-BREF* World Health Organization Quality of Life Questionnaire, *U* Mann–Whitney *U* test.

*Significant differences between groups with *p* < 0.05.

^a^*n* = 1 patient chose to make no specification.

#### Clinical symptoms

**Fig. 5:**
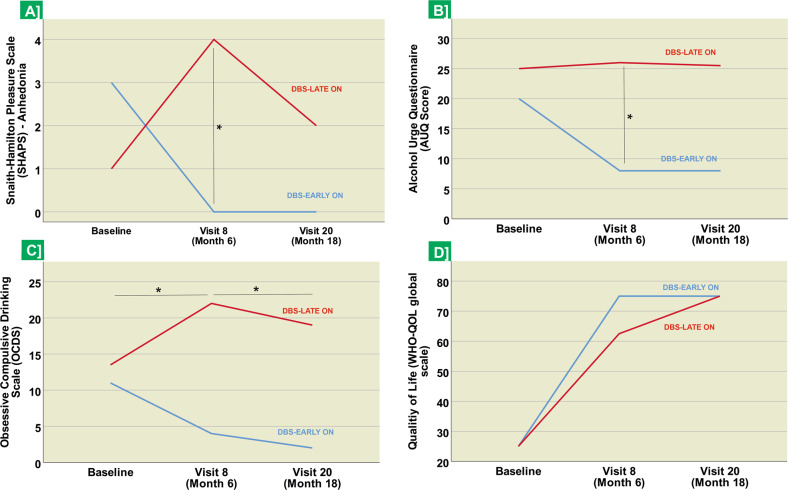
Effects of NAc DBS on anhedonia, alcohol craving and quality of life. Depiction of median values and of significant longitudinal changes and group differences in **A** anhedonia (SHAPS), **B**, **C** alcohol craving (OCDS and AUQ), and **D** quality of life (WHOQOL-BREF) at baseline, at the 6-month visit (i.e., end of blinded phase), and at 18 months (after all patients had been actively stimulated for at least 12 months). *Significant differences between time points or study groups, as indicated, at *p* < 0.05, determined using Wilcoxon tests and Mann–Whitney *U* tests.

The DBS-EARLY ON group reported significantly lower alcohol craving at the end of the blinded study phase, during study visit 8 (AUQ score, *p* = 0.020, see Table 2A and Fig. 5) and lower anhedonia scores on two scales (SHAPS, *p* = 0.028, and the Chapman Anhedonia scale, *p* = 0.008) compared to the DBS-LATE ON group. Longitudinal analyses across both groups showed a significant reduction in alcohol craving (OCDS) from baseline to month 6 (*n* = 10, baseline: 21.0 ± 7.0, 6 M: 10.5 ± 7.2 *Z* = −2,191, *p* = 0.014) and month 18 (*n* = 8, baseline: 21.0 ± 7.0, 18 M: 5.9 ± 6.4 *Z* = −2,371, *p* = 0.008). Mirroring these findings, we also found decreases in alcohol craving (AUQ) although these were not statistically significant (see Supplementary Table 2). We further observed a reduction in depressive symptoms (HAMD, BDI) and anhedonia (SHAPS) as well as increases in quality-of-life indices (WHOQOL-BREF) and global functioning (GAF) that were larger in the DBS-EARLY ON compared to the DBS-LATE ON group (see Supplementary Table 2). However, these descriptive differences were not statistically significant, most likely due to limited power.

#### Safety

Within the first 6 months, a total of 38 adverse events (AEs) occurred in 6 patients, including 24 AEs and 7 serious adverse events (SAEs) in 4 patients in the DBS-EARLY ON group and 14 AEs and 7 SAEs in 2 patients in the DBS-LATE ON group. During the following 12 months, a total of 82 AEs were recorded in 9 patients. Of all of the AEs, 68.9% were rated as mild to moderate. SAEs were mostly related to alcohol relapse and re-admission for detoxification, as any case of inpatient treatment had to be considered by law as SAE. Six AEs were related to a surgical procedure (DBS-EARLY ON: *n* = 5; DBS-LATE ON: *n* = 1). Some AEs were related to the stimulation device itself: in one case, the AE was interpreted to be causally related to the stimulation device (premature battery depletion), while in 9 cases the stimulation device was considered as possibly related to the AE (for details see Supplementary Table 3). One patient experienced a premature battery depletion 11 months after randomization, leading to the replacement of the pulse generator. During the first 6 study months, 26 AEs required treatment, while 65 AEs required treatment during the following 12 months. Of AEs occurring within the first six study months, 85.7% were resolved, compared to 88.9% during the following 12 months. Within the 18-month study period, no deaths or lasting disabilities were reported. Both study groups (DBS-EARLY ON and DBS-LATE ON) did not differ significantly on the Young Mania rating scale (range 0–60) at any time point throughout the study period with scores ranging from 0 to a maximum of 7 points (*p*_min_ ≥ 0.690). In addition, the scores on the Young Mania rating scale across both groups did not show a significant increase or decrease over time (*p* = 0.301), suggesting absence of clinically relevant (hypo-)mania in the current sample.

### Exploratory responder analyses

Based on the trajectories of their alcohol use, three patients were classified as responders (i.e. reduction of mean alcohol use by two or more WHO risk drinking levels) and 8 as non-responders. The mean reduction in daily alcohol use from baseline to the last study visit (month 18) was 43.4 g/d (±226.7) in non-responders and 198.1 g/d (±52.7) in responders. At baseline, responders showed a significantly higher craving for alcohol (*p* = 0.042), significantly more depressive symptoms (*p* = 0.018), anxiety (*p* = 0.042) as well as higher anhedonia scores (*p* = 0.036, see Supplementary Table 4).

## Discussion

We present the first double-blind RCT investigating the effects of DBS on abstinence and alcohol use in AUD patients. The primary intention-to-treat analysis suggested an abstinence-promoting effect of DBS. However, the results were not statistically significant, most likely due to limited power resulting from the small sample size and the categorial primary outcome. Descriptively, the observed HR of 0.73 would translate to a NNT of 9.6, which – in the light of the NNTs observed for other relapse-preventing treatments and the sample of treatment resistant patients – is suggestive of a potential effect of DBS in AUD [55–57]. Obviously, the significance and robustness of this effect would need to be confirmed in larger samples. Further evidence for an abstinence-promoting effect was provided by the significant effects of active DBS (DBS-EARLY ON) on secondary parametric outcomes. Considering alcohol use at 6 months after randomization, the proportion of abstinent days for the DBS-EARLY ON group was significantly higher than that of the DBS-LATE ON group. In addition, the DBS-EARLY ON group reported fewer heavy drinking days. These findings are in line with preclinical animal models, which demonstrated significant effects of NAc DBS on alcohol use [25, 26]. Following case reports and a recent open-label trial in six patients investigating NAc DBS reported noteworthy abstinence rates and reductions of alcohol craving for patients with treatment-resistant AUD [27, 29, 32, 58, 59] and other substance-use disorders (for review see [60]). In the current trial, we observed a prominent decrease in alcohol use within two weeks of activated DBS in the DBS-EARLY ON group (see Supplementary Fig. 2). A similar effect also occurred in the DBS-LATE ON group receiving sham stimulation. These observations are in line with DBS studies in depression [61–63], obsessive compulsive disorder [64] and Parkinson’s disease [65] that reported significant non-stimulation-related effects of DBS on clinical symptoms in the sham stimulation groups early after onset of the intervention. It has been suggested that placebo effects, motivational effects and micro-lesion effects (due to electrode insertion, microtrauma and local edema without active stimulation) may account for these observations. Regarding the effects of micro-lesioning, animal models demonstrated that DBS induced neuro-inflammation at the target site and data in humans showed that acute antidepressant effects were reduced in those patients taking anti-inflammatory medication after surgery, linking non-stimulation-related effects of DBS to local inflammation [66]. In addition, it can be speculated that local electrode insertion at the NAc might lead to transient silencing of adjacent neurons, which might contribute to the effects, which we observed in the DBS-LATE ON group. Beyond that, placebo and motivational effects could have contributed to the acute effects observed in both groups. Even though the relative contribution of the different non-stimulation-related effects of DBS cannot be disentangled based on current data, any putative placebo effect would likely explain short-term effects but not long-term DBS effects as observed in our study. Significant differences between the two DBS groups emerged 5 months after randomization (see Supplementary Fig. 2) and alcohol use was significantly attenuated in the DBS-LATE ON group after stimulation was activated in the open-label phase (month 6–18). These observations indicate stimulation-specific effects of DBS on alcohol use and support the potential beneficial effects of NAc DBS in treatment resistant alcohol dependence.

In addition, we observed alcohol craving – a hallmark symptom of AUD that can act as a trigger for alcohol use – to be significantly reduced in the DBS-EARLY ON group after 6 months [67]. Even beyond the initial 6-month follow-up period, our data show a continued significant reduction in alcohol craving up to the end of the study, indicating that DBS might exert at least a part of its abstinence-promoting effects by attenuating alcohol craving. This finding suggests that patients experiencing high craving for alcohol might particularly benefit from NAc DBS treatment. Our exploratory responder analyses support this speculation by demonstrating that responders to DBS treatment were the ones exhibiting high alcohol craving at baseline. Positive effects of DBS on alcohol craving are in line with previous research investigating DBS in AUD [27, 29, 31] and other substance-use disorders [68–71].

Our responder analyses also indicated that responders to NAc DBS are those with higher depressiveness, anxiety and anhedonia scores at baseline. NAc DBS resulted in significantly lower anhedonia scores in the DBS-EARLY ON group versus DBS-LATE ON group. It is conceivable that the effects of DBS on alcohol use are also mediated in part by its positive impact on anhedonia, a common symptom in depression and anxiety, as well as in addiction. The positive effect of NAc DBS on anhedonia might also – at least in part – explain the observed positive effects on alcohol use during the open-label phase of the trial, when alcohol craving (OCDS and AUQ) did not show significant decreases, while alcohol use and anhedonia were attenuated. This finding is interesting in several ways: The NAc is considered a key brain region for regulating behaviors related not only to addiction, but also to depression. Previous studies in depressed patients reported significant effects of NAc DBS on depressive symptoms, including anhedonia [72–74]. Furthermore, neuroimaging studies in AUD showed that alcohol-cue-induced brain response in the NAc and the striatum can predict both relapse risk [16] as well as treatment response to naltrexone [18, 19].

Regarding the safety of DBS, we did not observe any adverse events or serious adverse events resulting in lasting disability or death over the 18-month trial period, and most AEs were mild to moderate. This is in line with previous research on DBS [60]. The available data underscore the importance of carefully balancing the risks of DBS against the risks of uncontrolled alcohol use in treatment-resistant AUD. Future research could focus on investigating patients with comorbid depression and addiction. For this population, the combined morbidity and mortality risks resulting from treatment-resistant depression and addiction are substantial, supporting demand for new unconventional treatment approaches.

### Strengths and limitations

The presented results have to be considered against the backdrop of the strengths and limitations of previously published work. Due to the strict inclusion and exclusion criteria, the study was unable to yield the expected sample size of 30 patients in the allotted time. Such a sample size would have been necessary to provide sufficient statistical power for the primary intention-to-treat analysis which was not statistically significant, most likely due to the limited power originating from the small sample size and the categorial nature of the primary outcome parameter. Post-hoc estimates of statistical power indicated that the power to detect effect sizes corresponding to a HR of 0.73 was <15%. To illustrate the magnitude of the effect of DBS in treatment resistant AUD, we calculated the corresponding NNT, which indicated a medium effect of DBS in AUD. The wide confidence interval of the estimate for the NNT, however, indicates a high degree of uncertainty regarding the size of the true effect and should therefore be interpreted with caution. Inclusion of a parametric primary outcome could have provided higher power. Unfortunately, the use of such outcomes (e.g. mean alcohol use, proportion of heavy drinking days) had not yet been approved by the European Medicines Agency (EMA) when we planned and received funding for our study, and thus were not approved by the ethics boards. Future studies should consider outcomes beyond abstinence rates, such as number of heavy drinking days, which captured significant effects of NAc DBS in the current study. The sample of our study can still be considered as sizable in the light of the very low sample sizes of previous DBS AUD studies in the literature (range *n* = 1 to 6; for a review, see [60]). The start of the sham stimulation phase directly after surgery leaves open the possibility that non-stimulation-related effects (e.g. placebo effects and micro-lesioning effects), as well ongoing optimization of stimulation parameters in some patients might have contributed to the observed efficacy of DBS in the early trial phase. In addition, even though the targeting procedures were standardized across all patients and centers and a thorough review process of the implanted electrodes after the operation did not identify any major violations of the implantation protocol that would require repositing of electrodes, slight variations of the electrode positions between patients and centers cannot be ruled out. This, together with minimal changes of the stimulation parameters (3.5–4.5 V) during the early stimulation phase, may have resulted in stimulation of slightly different brain networks. While it was beyond the scope of the presented work, application of MRI-based tractography could contribute to the refinement of electrode placement and a better characterization of the stimulated networks in future studies that might also promote a better understanding of the observed variable treatment responses. Our exploratory responder analyses indicated that especially patients with higher scores in depression, anxiety and anhedonia might be those responding to DBS in treatment-resistant AUD. This conclusion however is limited by the low overall scores and the fact that the current study did not enroll patients meeting the diagnostic criteria of a depressive disorder or anxiety disorder.

## Conclusion

We report a prospective double-blind randomized controlled trial investigating DBS in treatment-resistant AUD patients. While the primary intention-to-treat analysis did not produce a statistically significant result, the findings are nonetheless suggestive of a beneficial effect of DBS. This conjecture is supported by significant effects of DBS on important secondary outcomes, including the proportion of abstinent days, heavy drinking days, alcohol craving and anhedonia during the blinded study phase. We were able to provide evidence for the specificity of the observed effects using a double-blind lead-in phase. Additionally, we conducted an exploratory responder analysis, which indicated that patients suffering from high craving, depression and anhedonia might stand to benefit particularly from DBS.

## Supplementary information

Supplements

## References

[1] World Health Organization. Global status report on alcohol and health 2018 Geneva: World Health Organization; 2018. Available at: https://www.who.int/substance_abuse/publications/global_alcohol_report/en/.

[2] Fleury MJ, Djouini A, Huỳnh C, Tremblay J, Ferland F, Ménard JM (2016). Remission from substance use disorders: a systematic review and meta-analysis. Drug Alcohol Depend.

[3] Luigjes J, Segrave R, de Joode N, Figee M, Denys D (2019). Efficacy of invasive and non-invasive brain modulation interventions for addiction. Neuropsychol Rev.

[4] Robinson MJ, Robinson TE, Berridge KC (2013). Incentive salience and the transition to addiction. Biol Res Addict.

[5] Koob GF, Volkow ND (2010). Neurocircuitry of addiction. Neuropsychopharmacology.

[6] Koob GF (2009). Dynamics of neuronal circuits in addiction: reward, antireward, and emotional memory. Pharmacopsychiatry.

[7] Koob GF, Volkow ND (2016). Neurobiology of addiction: a neurocircuitry analysis. Lancet Psychiatry.

[8] Volkow ND, Koob GF, McLellan AT (2016). Neurobiologic advances from the brain disease model of addiction. N Engl J Med.

[9] Volkow ND, Fowler JS, Wang GJ, Swanson JM, Telang F (2007). Dopamine in drug abuse and addiction: results of imaging studies and treatment implications. Arch Neurol.

[10] Volkow ND, Fowler JS, Wang GJ, Telang F, Logan J, Jayne M (2010). Cognitive control of drug craving inhibits brain reward regions in cocaine abusers. NeuroImage.

[11] Schacht JP, Anton RF, Myrick H (2013). Functional neuroimaging studies of alcohol cue reactivity: a quantitative meta-analysis and systematic review. Addict Biol.

[12] Yalachkov Y, Kaiser J, Naumer MJ (2012). Functional neuroimaging studies in addiction: multisensory drug stimuli and neural cue reactivity. Neurosci Biobehav Rev.

[13] Grüsser SM, Wrase J, Klein S, Hermann D, Smolka MN, Ruf M (2004). Cue-induced activation of the striatum and medial prefrontal cortex is associated with subsequent relapse in abstinent alcoholics. Psychopharmacology.

[14] Heinz A, Siessmeier T, Wrase J, Hermann D, Klein S, Grüsser SM (2004). Correlation between dopamine D(2) receptors in the ventral striatum and central processing of alcohol cues and craving. Am J Psychiatry.

[15] Kühn S, Gallinat J (2011). Common biology of craving across legal and illegal drugs – a quantitative meta-analysis of cue-reactivity brain response. Eur J Neurosci.

[16] Bach P, Vollstädt-Klein S, Kirsch M, Hoffmann S, Jorde A, Frank J (2015). Increased mesolimbic cue-reactivity in carriers of the mu-opioid-receptor gene OPRM1 A118G polymorphism predicts drinking outcome: a functional imaging study in alcohol dependent subjects. Eur Neuropsychopharmacol.

[17] Beck A, Wustenberg T, Genauck A, Wrase J, Schlagenhauf F, Smolka MN (2012). Effect of brain structure, brain function, and brain connectivity on relapse in alcohol-dependent patients. Arch Gen Psychiatry.

[18] Bach P, Weil G, Pompili E, Hoffmann S, Hermann D, Vollstadt-Klein S (2021). FMRI-based prediction of naltrexone response in alcohol use disorder: a replication study. Eur Arch Psychiatry Clin Neurosci.

[19] Mann K, Vollstadt-Klein S, Reinhard I, Lemenager T, Fauth-Buhler M, Hermann D (2014). Predicting naltrexone response in alcohol-dependent patients: the contribution of functional magnetic resonance imaging. Alcohol Clin Exp Res.

[20] Bach P, Weil G, Pompili E, Hoffmann S, Hermann D, Vollstadt-Klein S (2020). Incubation of neural alcohol cue reactivity after withdrawal and its blockade by naltrexone. Addict Biol.

[21] Jakobs M, Fomenko A, Lozano AM, Kiening KL (2019). Cellular, molecular, and clinical mechanisms of action of deep brain stimulation-a systematic review on established indications and outlook on future developments. EMBO Mol Med.

[22] Alonso P, Cuadras D, Gabriels L, Denys D, Goodman W, Greenberg BD (2015). Deep brain stimulation for obsessive-compulsive disorder: a meta-analysis of treatment outcome and predictors of response. PLoS ONE.

[23] Luigjes JV, Van Den Brink W, Feenstra MV, Van Den Munckhof P, Schuurman P, Schippers R (2012). Deep brain stimulation in addiction: a review of potential brain targets. Mol Psychiatry.

[24] Lüscher C, Pollak P (2016). Optogenetically inspired deep brain stimulation: linking basic with clinical research. Swiss Med Wkly.

[25] Henderson MB, Green AI, Bradford PS, Chau DT, Roberts DW, Leiter JC (2010). Deep brain stimulation of the nucleus accumbens reduces alcohol intake in alcohol-preferring rats. Neurosurg focus.

[26] Knapp CM, Tozier L, Pak A, Ciraulo DA, Kornetsky C (2009). Deep brain stimulation of the nucleus accumbens reduces ethanol consumption in rats. Pharmacol Biochem Behav.

[27] Kuhn J, Lenartz D, Huff W, Lee S, Koulousakis A, Klosterkoetter J (2007). Remission of alcohol dependency following deep brain stimulation of the nucleus accumbens: valuable therapeutic implications?. J Neurol Neurosurg Psychiatry.

[28] Müller U, Sturm V, Voges J, Heinze H-J, Galazky I, Büntjen L (2016). Nucleus accumbens deep brain stimulation for alcohol addiction–safety and clinical long-term results of a pilot trial. Pharmacopsychiatry.

[29] Müller U, Sturm V, Voges J, Heinze H-J, Galazky I, Heldmann M (2009). Successful treatment of chronic resistant alcoholism by deep brain stimulation of nucleus accumbens: first experience with three cases. Pharmacopsychiatry.

[30] Voges J, Mueller U, Bogerts B, Muente T, Heinze H-J (2013). Deep brain stimulation surgery for alcohol addiction. World Neurosurg.

[31] Kuhn J, Gründler TOJ, Bauer R, Huff W, Fischer AG, Lenartz D (2011). Successful deep brain stimulation of the nucleus accumbens in severe alcohol dependence is associated with changed performance monitoring. Addict Biol.

[32] Davidson B, Giacobbe P, George TP, Nestor SM, Rabin JS, Goubran M (2022). Deep brain stimulation of the nucleus accumbens in the treatment of severe alcohol use disorder: a phase I pilot trial. Mol Psychiatry.

[33] Garey LJ (1997). Atlas of the human brain. J Anat.

[34] Sturm V, Lenartz D, Koulousakis A, Treuer H, Herholz K, Klein JC (2003). The nucleus accumbens: a target for deep brain stimulation in obsessive-compulsive- and anxiety-disorders. J Chem Neuroanat.

[35] Schlaepfer TE, Cohen MX, Frick C, Kosel M, Brodesser D, Axmacher N (2008). Deep brain stimulation to reward circuitry alleviates anhedonia in refractory major depression. Neuropsychopharmacology.

[36] Denys D, Mantione M, Figee M, van den Munckhof P, Koerselman F, Westenberg H (2010). Deep brain stimulation of the nucleus accumbens for treatment-refractory obsessive-compulsive disorder. Arch Gen Psychiatry.

[37] Tonigan JS, Miller WR, Brown JM (1997). The reliability of Form 90: an instrument for assessing alcohol treatment outcome. J Stud Alcohol.

[38] Anton RF, Moak DH, Latham P (1995). The Obsessive Compulsive Drinking Scale: a self-rated instrument for the quantification of thoughts about alcohol and drinking behavior. Alcohol Clin Exp Res.

[39] Mann K, Ackermann K (2000). Die OCDS-G: psychometrische kennwerte der deutschen version der obsessive compulsive drinking scale. SUCHT.

[40] Bohn MJ, Krahn DD, Staehler BA (1995). Development and initial validation of a measure of drinking urges in abstinent alcoholics. Alcohol Clin Exp Res.

[41] Beck AT, Ward CH, Mendelson M, Mock J, Erbaugh J (1961). An inventory for measuring depression. Arch Gen Psychiatry.

[42] Hamilton M, Guy W (1976). Hamilton depression scale. Group.

[43] Franken IH, Rassin E, Muris P (2007). The assessment of anhedonia in clinical and non-clinical populations: further validation of the Snaith-Hamilton Pleasure Scale (SHAPS). J Affect Disord.

[44] Chapman LJ, Chapman JP, Raulin ML (1976). Scales for physical and social anhedonia. J Abnorm Psychol.

[45] Young RC, Biggs JT, Ziegler VE, Meyer DA (1978). A rating scale for mania: reliability, validity and sensitivity. Br J Psychiatry: J Ment Sci.

[46] World Health Organization. Division of Mental H. WHOQOL-BREF: Introduction, Administration, Scoring And Generic Version Of The Assessment: Field Trial Version, December 1996. Geneva: World Health Organization; 1996.

[47] Hall RC (1995). Global assessment of functioning: a modified scale. Psychosomatics.

[48] First M, Spitzer R, Gibbon M, Williams J. Structured clinical interview for DSM-IV axis I disorders, clinician version (SCID-CV). Washington, DC: American Psychiatric Press; 1997.

[49] Hare RD. The Hare Psychopathy Checklist - Revised. Toronto, Ontario: Multi-Health Systems; 1991.

[50] Mann K, Aubin HJ, Witkiewitz K (2017). Reduced drinking in alcohol dependence treatment, what is the evidence?. Eur Addict Res.

[51] Witkiewitz K, Falk DE, Litten RZ, Hasin DS, Kranzler HR, Mann KF (2019). Maintenance of World Health Organization risk drinking level reductions and posttreatment functioning following a large alcohol use disorder clinical trial. Alcohol: Clin Exp Res.

[52] Knox J, Scodes J, Witkiewitz K, Kranzler HR, Mann K, O’Malley SS (2020). Reduction in World Health Organization risk drinking levels and cardiovascular disease. Alcohol Clin Exp Res.

[53] Witkiewitz K, Hallgren KA, Kranzler HR, Mann KF, Hasin DS, Falk DE (2017). Clinical validation of reduced alcohol consumption after treatment for alcohol dependence using the World Health Organization risk drinking levels. Alcohol Clin Exp Res.

[54] Altman DG, Andersen PK. Calculating the number needed to treat for trials where the outcome is time to an event. BMJ. 319;1492-5:1999. 10.1136/bmj.319.7223.1492 PMC1117211 10582940

[55] Jonas DE, Amick HR, Feltner C, Bobashev G, Thomas K, Wines R (2014). Pharmacotherapy for adults with alcohol use disorders in outpatient settings: a systematic review and meta-analysis. J Am Med Assoc.

[56] Rosner S, Hackl-Herrwerth A, Leucht S, Lehert P, Vecchi S, Soyka M (2010). Acamprosate for alcohol dependence. Cochrane Database Syst Rev.

[57] Rosner S, Hackl-Herrwerth A, Leucht S, Vecchi S, Srisurapanont M, Soyka M (2010). Opioid antagonists for alcohol dependence. Cochrane Database Syst Rev.

[58] Voges J, Müller U, Bogerts B, Münte T, Heinze H-J (2013). Deep brain stimulation surgery for alcohol addiction. World Neurosurg.

[59] Müller UJ, Voges J, Steiner J, Galazky I, Heinze HJ, Möller M (2013). Deep brain stimulation of the nucleus accumbens for the treatment of addiction. Ann N Y Acad Sci.

[60] Hassan O, Phan S, Wiecks N, Joaquin C, Bondarenko V (2021). Outcomes of deep brain stimulation surgery for substance use disorder: a systematic review. Neurosurg Rev.

[61] Coenen VA, Bewernick BH, Kayser S, Kilian H, Boström J, Greschus S (2019). Superolateral medial forebrain bundle deep brain stimulation in major depression: a gateway trial. Neuropsychopharmacology.

[62] Fenoy AJ, Schulz P, Selvaraj S, Burrows C, Spiker D, Cao B (2016). Deep brain stimulation of the medial forebrain bundle: Distinctive responses in resistant depression. J Affect Disord.

[63] Bewernick BH, Kayser S, Gippert SM, Coenen VA, Schlaepfer TE (2017). Acute antidepressant effects of deep brain stimulation–review and data from slMFB-stimulation. Personalized Med Psychiatry.

[64] Schruers K, Baldi S, van den Heuvel T, Goossens L, Luyten L, Leentjens AFG (2019). The effects of deep-brain non-stimulation in severe obsessive-compulsive disorder: an individual patient data meta-analysis. Transl Psychiatry.

[65] Tykocki T, Nauman P, Koziara H, Mandat T (2013). Microlesion effect as a predictor of the effectiveness of subthalamic deep brain stimulation for Parkinson’s disease. Stereotact Funct Neurosurg.

[66] Perez-Caballero L, Perez-Egea R, Romero-Grimaldi C, Puigdemont D, Molet J, Caso J (2014). Early responses to deep brain stimulation in depression are modulated by anti-inflammatory drugs. Mol Psychiatry.

[67] Murphy CM, Stojek MK, Few LR, Rothbaum AO, Mackillop J (2014). Craving as an alcohol use disorder symptom in DSM-5: an empirical examination in a treatment-seeking sample. Exp Clin Psychopharmacol.

[68] Ge S, Chen Y, Li N, Qu L, Li Y, Jing J (2019). Deep brain stimulation of nucleus accumbens for methamphetamine addiction: two case reports. World Neurosurg.

[69] Valencia-Alfonso C-E, Luigjes J, Smolders R, Cohen MX, Levar N, Mazaheri A (2012). Effective deep brain stimulation in heroin addiction: a case report with complementary intracranial electroencephalogram. Biol Psychiatry.

[70] Mantione M, van de Brink W, Schuurman PR, Denys D (2010). Smoking cessation and weight loss after chronic deep brain stimulation of the nucleus accumbens: therapeutic and research implications: case report. Neurosurgery.

[71] Chen L, Li N, Ge S, Lozano AM, Lee DJ, Yang C (2019). Long-term results after deep brain stimulation of nucleus accumbens and the anterior limb of the internal capsule for preventing heroin relapse: an open-label pilot study. Brain Stimul.

[72] Bewernick BH, Hurlemann R, Matusch A, Kayser S, Grubert C, Hadrysiewicz B (2010). Nucleus accumbens deep brain stimulation decreases ratings of depression and anxiety in treatment-resistant depression. Biol Psychiatry.

[73] Xu L, Nan J, Lan Y (2020). The nucleus accumbens: a common target in the comorbidity of depression and addiction. Front Neural Circuits.

[74] Myrick H, Anton RF, Li X, Henderson S, Randall PK, Voronin K (2008). Effect of naltrexone and ondansetron on alcohol cue–induced activation of the ventral striatum in alcohol-dependent people. Arch Gen Psychiatry.

[75] Horn A, Li N, Dembek TA, Kappel A, Boulay C, Ewert S (2019). Lead-DBS v2: towards a comprehensive pipeline for deep brain stimulation imaging. NeuroImage.

[76] Edlow BL, Mareyam A, Horn A, Polimeni JR, Witzel T, Tisdall MD (2019). 7 Tesla MRI of the ex vivo human brain at 100 micron resolution. Sci Data.

