# Supplementary material for: Deep brain stimulation of the nucleus accumbens in treatment-resistant alcohol use disorder: a double-blind randomized controlled multi-center trial
Source: Transl Psychiatry. 2023 Feb 8;13:49. doi: 10.1038/s41398-023-02337-1 (PMC9908935; doi:10.1038/s41398-023-02337-1)
Supplement: Supplementary file 1 — Supplements [file 41398_2023_2337_MOESM1_ESM.docx]

**Supplements**

*Deep brain stimulation of the nucleus accumbens in treatment-resistant alcohol-dependent patients: a double-blind randomized controlled multi-center trial*

**Content:**

- **Supplementary Tables**
  - **Supplementary Table 1.** Indirect alcohol use markers showed a decrease over time in both study groups.
  - **Supplementary Table 2.** Depiction of mean values for psychometric measures of depression, anxiety, anhedonia and quality of life at baseline and visit 20 (18 months after randomization) for both study groups.
  - **Supplementary Table 3**. Adverse Events that were deemed to be related or possibly related to the DBS intervention.
  - **Supplementary Table 4.** Baseline demographic data, alcohol use and severity measures for responders and non-responders at baseline
  - **Supplementary Table 5.** Depiction of individual patients values for the primary outcome and key secondary outcomes at study visit 8, i.e. 6 months after randomization, for all N=12 patients
- **Supplementary Figures**
  - **Supplementary Figure 1.** Depiction of the median proportion of drinking days across the study duration

**Supplementary Tables**

**Supplementary Table S1.** Indirect alcohol use markers showed a decrease over time in both study groups. The mean corpuscular volume (MCV) and Carbohydrate-deficient transferrin (CDT) showed a significant decrease from baseline to visit 8 (month 6 after randomization) in the whole sample. Separate longitudinal analyses within both groups did not reveal any significant differences over time (not shown here).

|  |  | **Baseline** | | **Visit 8**  **after 6 Months** | | **Visit 20**  **after 18 Months** |
| --- | --- | --- | --- | --- | --- | --- |
|  |  | **DBS-EARLY ON** | **DBS-LATE ON** | **DBS-EARLY ON** | **DBS-LATE ON** |  |
| **ASAT**  **(µmol/s.L)** | n  Mean ± SD  Median | 5  0.71±0.25  0.65 | 5  1.12±0.67  1.38 | 5  1.16±1.59  0.52 | 5  0.97±1.10  0.42 | 6  0.76±0.98  0.36 |
|  | Mann- Whitney Test | n.s. | | n.s. | | - |
|  | Wilcoxon Test (Baseline vs. other Visits) | - | | n.s. | | n.s. |
| **ALAT**  **(µmol/s.L)** | n  Mean ± SD  Median | 5  0.65±0.32  0.52 | 5  0.73±0.56  0.62 | 5  0.78±0.74  0.49 | 5  0.76±0.52  0.45 | 7  0.62±0.42  0.40 |
|  | Mann- Whitney Test | n.s. | | n.s. | | - |
|  | Wilcoxon Test (Baseline vs. other Visits) | - | | n.s. | | n.s. |
| **GGT (µmol/s.L)** | n  Mean ± SD  Median | 5  1.84±1.23  1.67 | 5  3.39±3.74  1.93 | 5  6.31±11.82  0.65 | 5  4.62±7.57  1.39 | 7  2.05±2.58  0.70 |
|  | Mann- Whitney Test | n.s. | | n.s. | | - |
|  | Wilcoxon Test (Baseline vs. other Visits) | - | | n.s. | | n.s. |
| **CDT (%)** | n  Mean ± SD  Median | 5  2.62±1.12  2.00 | 3  4.83±2.36  4.83 | 5  1.99±0.77  2.06 | 3  4.01±3.56  2.38 | 6  1.68±0.65  1.46 |
|  | Mann- Whitney Test | n.s. | | n.s. | | - |
|  | Wilcoxon Test (Baseline vs. other Visits) | - | | Z = 1.685, *p* = 0.05* | | n.s. |
| **MCV (fl)** | n  Mean ± SD  Median | 5  94.6±7.9  93.0 | 5  92.8±10.4  91.0 | 5  92.0±8.8  95.0 | 5  91.6±7.9  89.0 | 7  90.4±11.1  90.0 |
|  | Mann- Whitney Test | n.s. | | n.s. | | - |
|  | Wilcoxon Test (Baseline vs. other Visits) | - | | *Z* = 1.690, *p* = 0.05* | | n.s. |

ALAT = Alanin-Amino-Transferase; ASAT = Aspartate aminotransferase; CDT = Carbohydrate-deficient transferrin; GGT = Gamma-glutamyl transferase; MCV = Mean Corpuscular Volume; * = significant differences at *p* < 0.05; n.s. = not significant.

**Supplementary Table S2.** Depiction of mean values for psychometric measures of depression, anxiety, anhedonia and quality of life at baseline and visit 20 (18 months after randomization) for both study groups. Significant changes over time were found for alcohol craving (OCDS) from baseline to month 6 (*n* = 10, baseline: 21.0±7.0, 6-month visit: 10.5±7.2, *Z* = -2,191, *p* = 0.014) and month 18 (*n* = 8, baseline: 21.0±7.0, 18-month visit: 5.9±6.4, *Z* = -2,371, *p* = 0.008).

| Visit | **OCDS** | | **AUQ** | | **HAMD** | | **HAM-A** | | **BDI-II** | | **SHAPS** | | **Physical Anhedonia** | | **Social Anhedonia** | | **WHOQOL-BREF** | | **GAF** | | |
| --- | --- | --- | --- | --- | --- | --- | --- | --- | --- | --- | --- | --- | --- | --- | --- | --- | --- | --- | --- | --- | --- |
|  | 1 | 20 | 1 | 20 | 1 | 20 | 1 | 20 | 1 | 20 | 1 | 20 | 1 | 20 | 1 | 20 | 1 | 20 | 1 | 20 | |
| DBS-EARLY ON | | | | | | | | | | | | | | | | | | | | |  |
| Mean | 19.5 | 9.8 | 23.7 | 8.3 | 3.0 | 1.0 | 0.7 | 4.0 | 10.8 | 3.8 | 2.8 | 0.8 | 5.8 | 5.8 | 10.0 | 9.3 | 25.5 | 54.2 | 57.7 | 71.7 | |
| N | 4.0 | 4.0 | 3.0 | 3.0 | 3.0 | 3.0 | 3.0 | 3.0 | 4.0 | 4.0 | 4.0 | 4.0 | 4.0 | 4.0 | 4.0 | 4.0 | 3.0 | 3.0 | 3.0 | 3.0 | |
| SD | 5.2 | 10.3 | 15.8 | 0.6 | 5.2 | 1.0 | 1.2 | 4.0 | 4.0 | 6.8 | 1.3 | 1.5 | 2.9 | 2.8 | 6.8 | 3.7 | 11.8 | 36.1 | 15.4 | 26.6 | |
| DBS-LATE ON | | | | | | | | | | | | | | | | | | | | |  |
| Mean | 20.5 | 19.8 | 26.3 | 27.8 | 1.0 | 8.3 | 0.7 | 4.7 | 17.7 | 9.7 | 2.3 | 3.7 | 6.7 | 7.7 | 10.7 | 10.7 | 33.3 | 62.5 | 57.0 | 54.5 | |
| N | 4.0 | 4.0 | 4.0 | 4.0 | 3.0 | 3.0 | 3.0 | 3.0 | 3.0 | 3.0 | 3.0 | 3.0 | 3.0 | 3.0 | 3.0 | 3.0 | 3.0 | 3.0 | 4.0 | 4.0 | |
| SD | 9.1 | 14.7 | 18.3 | 23.1 | 1.0 | 13.6 | 0.6 | 5.0 | 9.0 | 10.0 | 2.3 | 4.7 | 0.6 | 0.6 | 2.1 | 5.1 | 26.0 | 21.7 | 12.6 | 22.8 | |

SD = Standard Deviation; DBS-EARLY ON = study group with active stimulation during the first six study months, DBS-LATE ON = group with sham stimulation during the first six study months; ADS = Alcohol Dependence Scale; AUQ = Alcohol Urge Questionnaire; AUDIT = Alcohol Use Disorders Identification Test; BDI-II = Beck Depression Inventory; GAF = Global Assessment of Functioning Scale; HAMD = Hamilton Depression Scale; HAM-A = Hamilton Anxiety Rating Scale; OCDS = Obsessive-Compulsive Drinking Scale; SHAPS = Snaith-Hamilton Pleasure Scale; STAI = State-Trait-Anxiety Inventory; SD = standard deviation; WHOQOL-BREF = World Health Organization Quality of Life Questionnaire

**Supplementary Table S3**. Adverse Events that were deemed to be related or possibly related to the DBS intervention.

| **Description of Adverse Event** | **Measure taken** | **Causal relation to stimulation** |
| --- | --- | --- |
| Premature battery depletion | Change of battery | Yes |
| Lack of drive, increased sleepiness | Adjustment of stimulation parameters | Possible |
| Device turned off at the request of the participant | Device turned off | Possible |
| Relapse during inpatient treatment with pathological intoxication | Transfer to closed ward, continuous monitoring, detoxification | Possible |
| Decreased libido, erectile dysfunction | - | Possible |
| Depressive syndrome | - | Possible |
| Difficulty staying asleep | Doxepin 100mg/d as needed | Possible |
| Headache | - | Possible |
| Loss of libido | - | Possible |
| Difficulty falling asleep and staying asleep | Psychoeducation on sleep hygiene | Possible |

**Supplementary Table S4.** Baseline demographic data, alcohol use and severity measures for responders and non-responders at baseline.

|  | **Non-Responders (n=8)** | **Responders (n=3)** | **Statistics** | **Significance** |
| --- | --- | --- | --- | --- |
| *Clinical scales* | Mean (SD) | Mean (SD) |  |  |
| AUDIT (sumscore) | 31.4 (3.9) | 35.3 (4.0) | *U* = 5.5 | *p* = 0.121 |
| ADS (sumscore) | 22.4 (9.7 | 21.7 (5.1) | *U* = 10.5 | *p* = 0.412 |
| OCDS (sumscore) | 20.0 (7.8) | 24.3 (8.0) | *U* = 7 | *p* = 0.188 |
| AUQ (sumscore) | 16.1 (10.7) | 34.0 (17.6 | *U* = 3 | *p* = 0.042* |
| FTND (sumscore)° | 6.1 (3.0) | 6.0 (3.0) | *U* = 9.5 | *p* = 0.467 |
| HAMD (sumscore) | 2.4 (3.9 | 0.7 (1.2) | *U* = 9 | *p* = 0.303 |
| BDI-II (sumscore) | 7.3 (4.3) | 19.0 (7.2) | *U* = 1.5 | *p* = 0.018* |
| SHAPS (sumscore) | 3.7 (3.5) | 3.0 (2.0) | *U* = 12 | *p* = 0.527 |
| Physical Anhedonia (sumscore) | 5.6 (4.1) | 8.0 (1.7) | *U* = 3 | *p* = 0.036* |
| Social Anhedonia (sumscore) | 9.3 (4.9) | 13.0 (6.1) | *U* = 6 | *p* = 0.139 |
| HAM-A (sumscore) | 3.3 (3.4) | 0.3 (0.6) | *U* = 2.5 | *p* = 0.042* |
| WHOQOL-BREF (global domain) | 53.8 (25.2) | 25.0 (12.5) | *U* = 4.5 | *p* = 0.079 |
| GAF (sumscore) | 51.0 (12.7) | 63.7 (7.5) | *U* = 4 | *p* = 0.067 |

ADS = Alcohol Dependence Scale; AUQ = Alcohol Urge Questionnaire; AUDIT = Alcohol Use Disorders Identification Test; BDI-II = Beck Depression Inventory; FTND = Fagerstroem Test for Nicotine Dependence; GAF = Global Assessment of Functioning Scale; HAMD = Hamilton Depression Scale; HAM-A = Hamilton Anxiety Rating Scale; OCDS = Obsessive-Compulsive Drinking Scale; SHAPS = Snaith-Hamilton Pleasure Scale; STAI = State-Trait-Anxiety Inventory; SD = standard deviation; WHOQOL-BREF = World Health Organization Quality of Life Questionnaire; * = significant differences between groups with *p* < 0.05; ° *n* = 1 patient chose to make no specification.

| **ID** | **Treatment** | **Center** | **Relapse (within first 6 months)** | **Time to Relapse (days)** | **Ethanol**  **(g/day; mean of last 30 days)** | **Drinks per day**  **(à 12g, mean of last 30 days)** | **Abstinent days**  **(in last 30 days)** | **Heavy Drinking days (>60g/d in last 30 days)** | **OCDS**  **(sumscore)** | **AUQ (sumscore)** |
| --- | --- | --- | --- | --- | --- | --- | --- | --- | --- | --- |
| 1 | DBS-EARLY ON | Magdeburg | Yes | 10 | 4909.09 | 14 | 18.18% | 24.55% | 9 | 14 |
| 2 | DBS-EARLY ON | Magdeburg | NO | 244 | 0 | 0 | 100.00% | 0 | 2 | 8 |
| 3 | DBS-EARLY ON | Cologne | YES | 153 | 580.65 | 2 | 67.74% | 9.68% | 1 | 8 |
| 4 | DBS-EARLY ON | Cologne | YES | 7 | - | - | - | - | - | - |
| 5 | DBS-EARLY ON | Mannheim | YES | 3 | 5154.00 | 14 | 72.00% | 7.2% | 16 | 9 |
| 6 | DBS-EARLY ON | Mannheim | YES | 6 | 146.34 | 0 | 97.56% | 0.73% | 12 | 17 |
| 7 | DBS-LATE ON | Magdeburg | YES | 79 | 2142.86 | 6 | 64.29% | 10.71% | 24 | 14 |
| 8 | DBS-LATE ON | Magdeburg | YES | 33 | - | - | - | - | - | - |
| 9 | DBS-LATE ON | Cologne | YES | 14 | - | - | 46.43% | 16.07% | 28 | 15 |
| 10 | DBS-LATE ON | Cologne | YES | 7 | 2640.00 | 7 | 20.00% | 24.00% | 21 | 32 |
| 11 | DBS-LATE ON | Mannheim | YES | 2 | 2396.25 | 7 | 59.38% | 12.19% | 32 | 20 |
| 12 | DBS-LATE ON | Mannheim | YES | 43 | 5057.14 | 14 | 14.29% | 22.86% | 7 | 36 |

**Supplementary Table 5.** Depiction of individual values for the primary outcome and key secondary outcomes at study visit 8, i.e. 6 months after randomization, for all N=12 patients

AUQ = Alcohol Urge Questionnaire; OCDS = Obsessive-Compulsive Drinking Scale; Missing values are indicated by “-“.

**Supplementary Figures**

**Supplementary Figure 1.** Depiction of the median proportion of drinking days across the study duration (baseline until end of the blinded study period) for both study groups. * = significant differences between groups at specific time points. Initially, a drop in the proportion of drinking days was observed in both study groups.
